# The effect of simvastatin on lipid droplets accumulation in human embryonic kidney cells and pancreatic cancer cells

**DOI:** 10.1186/1476-511X-12-126

**Published:** 2013-08-21

**Authors:** Helena Gbelcová, Martin Švéda, Lucia Laubertová, Ivan Varga, Libor Vítek, Michal Kolář, Hynek Strnad, Jaroslav Zelenka, Daniel Böhmer, Tomáš Ruml

**Affiliations:** 1 Institute of Medical Biology, Genetics and Clinical Genetics, Faculty of Medicine, Comenius University, Bratislava, Slovakia; 2 Department of Biochemistry and Microbiology, Institute of Chemical Technology, Prague, Czech Republic; 3 Institute of Medical Biochemistry, Jessenius Faculty of Medicine, Comenius University, Martin, Slovakia; 4 Institute of Histology and Embryology, Faculty of Medicine, Comenius University, Bratislava, Slovakia; 5 Institute of Medical Biochemistry and Laboratory Diagnostics, and 4th Department of Internal Medicine, 1st Faculty of Medicine, Charles University in Prague, Prague, Czech Republic; 6 Laboratory of Genomics and Bioinformatics, Institute of Molecular Genetics, Czech Academy of Sciences, Prague, Czech Republic; 7 Institute of Medical Biochemistry and Laboratory Diagnostics, 1st Faculty of Medicine, Charles University in Prague, Prague, Czech Republic; 8 Current affiliation: Institute of Physiology, Czech Academy of Sciences, Prague, Czech Republic

**Keywords:** Simvastatin, Lipid droplets, DNA microarray, Nile red, Pancreatic cancer

## Abstract

**Background:**

Statins (HMG-CoA reductase inhibitors) represent a major class of compounds for the treatment of hypercholesterolemia due to their ability to inhibit *de novo* cholesterol synthesis. In addition to their hypolipidemic effects, chemoprotective properties have been attributed to statins as well. These effects involve multiple mechanisms, which, however, are not known in detail. The aim of our study was to assess in non-malignant as well as cancer cells the impact of simvastatin on the amount of cytosolic lipid droplets (LDs) implicated in many biological processes including proliferation, inflammation, carcinogenesis, apoptosis, necrosis or growth arrest.

**Methods:**

Human embryonic kidney cells HEK-293T and human pancreatic cancer cells MiaPaCa-2 were treated with simvastatin (6 and 12 μM) for 24 and 48 hours respectively. Neutral lipid probe Nile Red was used for detection of LDs by fluorescence microscopy. Cellular cholesterol content was determined by HPLC. Changes in expression of genes related to lipid metabolism in simvastatin-treated MiaPaCa-2 cells were examined by DNA microarray analysis. Validation of gene expression changes was performed using quantitative RT-PCR.

**Results:**

The treatment of the cells with simvastatin increased their intracellular content of LDs in both non-malignant as well as cancer cells, partially due to the uptake of cholesterol and triacylglyceroles from medium; but in particular, due to enhanced synthesis of triacylglyceroles as proved by significant overexpression of genes related to *de novo* synthesis of triacylglyceroles and phospholipids. In addition, simvastatin also markedly influenced expression of genes directly affecting cell proliferation and signaling.

**Conclusions:**

Simvastatin treatment led to accumulation of cytosolic LDs within the examined cells, a phenomenon which might contribute to the antiproliferative effects of statins.

## Background

Statins represent a major class of drugs for treatment of hypercholesterolemia due to their ability to inhibit *de novo* cholesterol synthesis, namely the rate-limiting step of mevalonate pathway catalyzed by HMG-CoA reductase. The first statin was discovered in 1976, and since 1980 they have been introduced into clinical practice [1].

In addition to their hypolipidemic effects, statins exert also numerous additional biological activities mediated by various products of the mevalonate pathway, such as anti-inflammatory and immunomodulatory activities, effects on endothelial function, anti-oxidative effects, or effects on cell proliferation and apoptosis, etc. [2,3].

Chemoprotective effects of statins have been attributed to their impact on protein prenylation. Among prenylated proteins, the low molecular weight GTP-binding Ras proteins constitute central regulators of numerous cellular functions including cell proliferation. In normal, untransformed cells, the Ras proteins cycle between an inactive GDP-bound state and active GTP-bound state at the plasma membrane (PM) [4]. Unlike activation mechanisms under physiological conditions, activating mutations of *ras* genes (mostly in *K-ras* gene) occur frequently in malignant tissues.

These mutations result in a loss of GTPase activity, which leads to permanent K-Ras protein activation and continuous initiation of downstream signal cascades related to cell proliferation. This results in deregulated growth of cell populations and development of cancer. It was found that approximately 30% of all human cancers harbor activating mutations of *ras* genes. In pancreatic cancer, these mutations of the *K-ras* oncogene are present in up to 90% of cases [5,6]. Statin-mediated inhibition of Ras protein farnesylation thus seems to be a promising adjuvant approach for the treatment of pancreatic cancer and cancer in general, and this is why the possible anticancer potential of statins is being intensively studied [7-9].

The molecular mechanisms of the anticancer effects of statins are complex and depend on many factors affecting their pharmacokinetics and pharmacodynamics [9,10]. Apart from common cellular processes affected by statins, their impact on metabolism of cytosolic lipid droplets (LDs) has not been studied in detail. Cytosolic LDs, previously considered to be passive fat deposits within the cells, are being currently regarded as dynamic, regulated organelles with multiple biological functions [11]. The mechanisms of LDs biogenesis and turnover are not currently understood and several hypotheses have been suggested [12,13]. LDs exist virtually in any kind of cell, ranging from bacteria to yeasts, plants, and higher mammals. In mammalian cells and in most cultured cell lines, LDs consist of a core of neutral lipids, predominantly triacylglyceroles (TG) or cholesteryl esters (CE), that are surrounded by a monolayer of phospholipids, free cholesterol and associated proteins. The neutral lipids that are stored in LDs are used for metabolism, membrane synthesis (phospholipids and cholesterol) and steroid synthesis [14]. In addition, LDs have a crucial role as a deposit of cholesterol in the form of cholesteryl esters, as part of complex homeostatic mechanisms that are involved in regulation of the intracellular free cholesterol levels [14]. Di Vito and co-workers reported a highly dynamic role of intracellular LDs in many important developmental processes including proliferation, inflammation, apoptosis, growth arrest and necrosis [15,16]. Based on these data, the effect of statins on pancreatic cancer cell proliferation might be caused by the changes in intracellular LDs amount.

The aim of the present study was thus to assess the effect of simvastatin on intracellular LDs in human healthy and pancreatic cancer cells, and to relate these effects to antiproliferative activities of statins.

## Results

### Analysis of intracellular LDs content

#### a) HEK 293T cells

**Figure 1:**
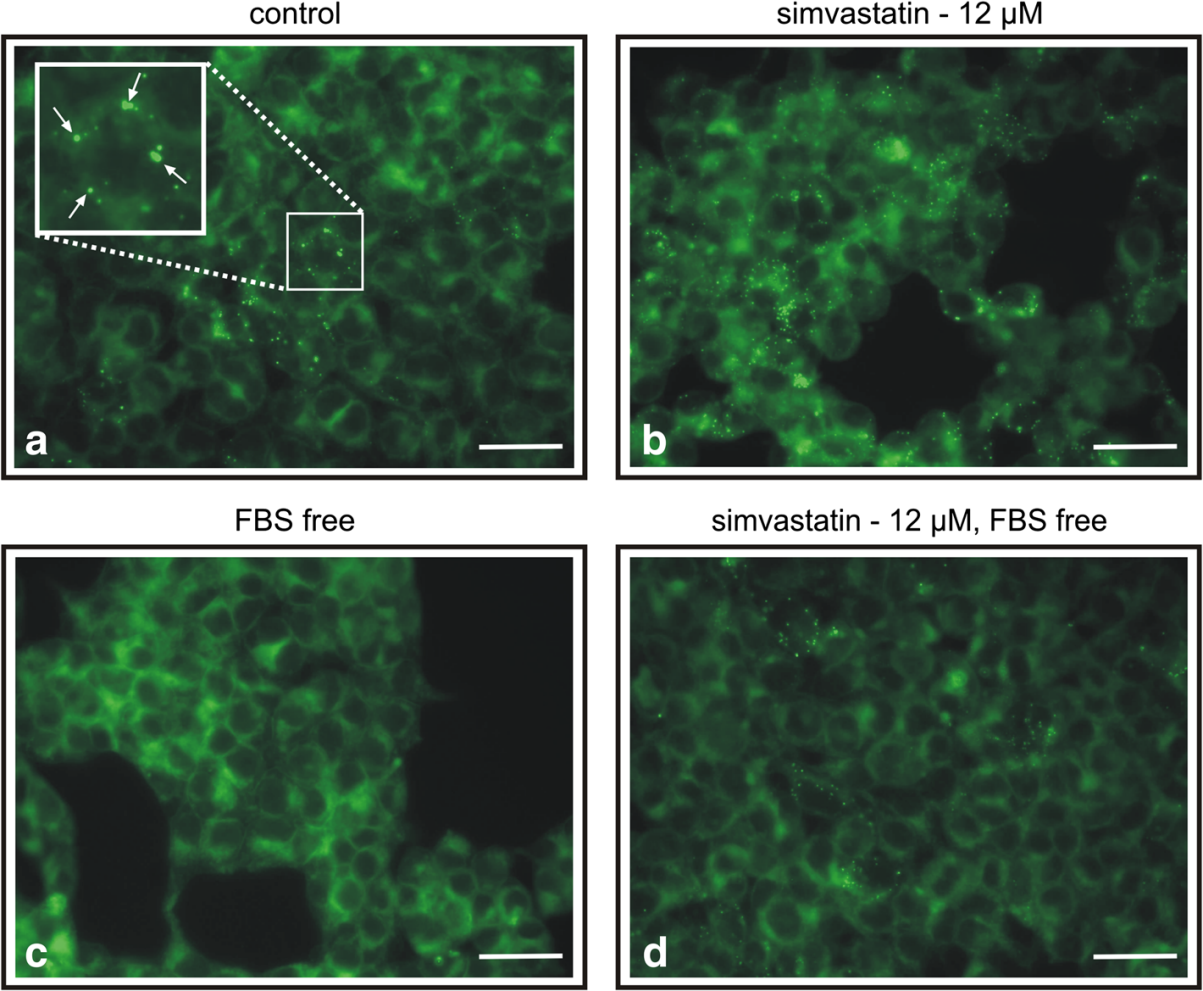
**Intracellular lipid droplets in HEK-293T cells staining by Nile Red (10 ng/ml), a) control cells, b) simvastatin treated cells, c) cells cultivated in FBS free medium, d) cells treated with simvastatin in FBS free medium.** Simvastatin used in concentration 12 μM, 24 hrs exposure. FBS, fetal bovine serum. Scale bar represents 10 μm. Arrows indicate lipid droplets.

Occasional presence of LDs was observed in HEK 293T cells cultured in the medium supplement with FBS (Figure 1a), which increased markedly in the cells exposed to simvastatin (12 μM) (Figure 1b). To eliminate the effect of possible cholesterol uptake from the medium, the cells were cultured in the FBS free medium. The absence of FBS resulted in disappearance of LDs from the cells not exposed to simvastatin (Figure 1c). However, compared to simvastatin-untreated cells (Figure 1c), the LDs content in the cells cultured in presence of 12 μM simvastatin in FBS free medium increased again (Figure 1d) and was comparable to the control cells (Figure 1a).

#### b) MiaPaCa-2 cells

**Figure 2:**
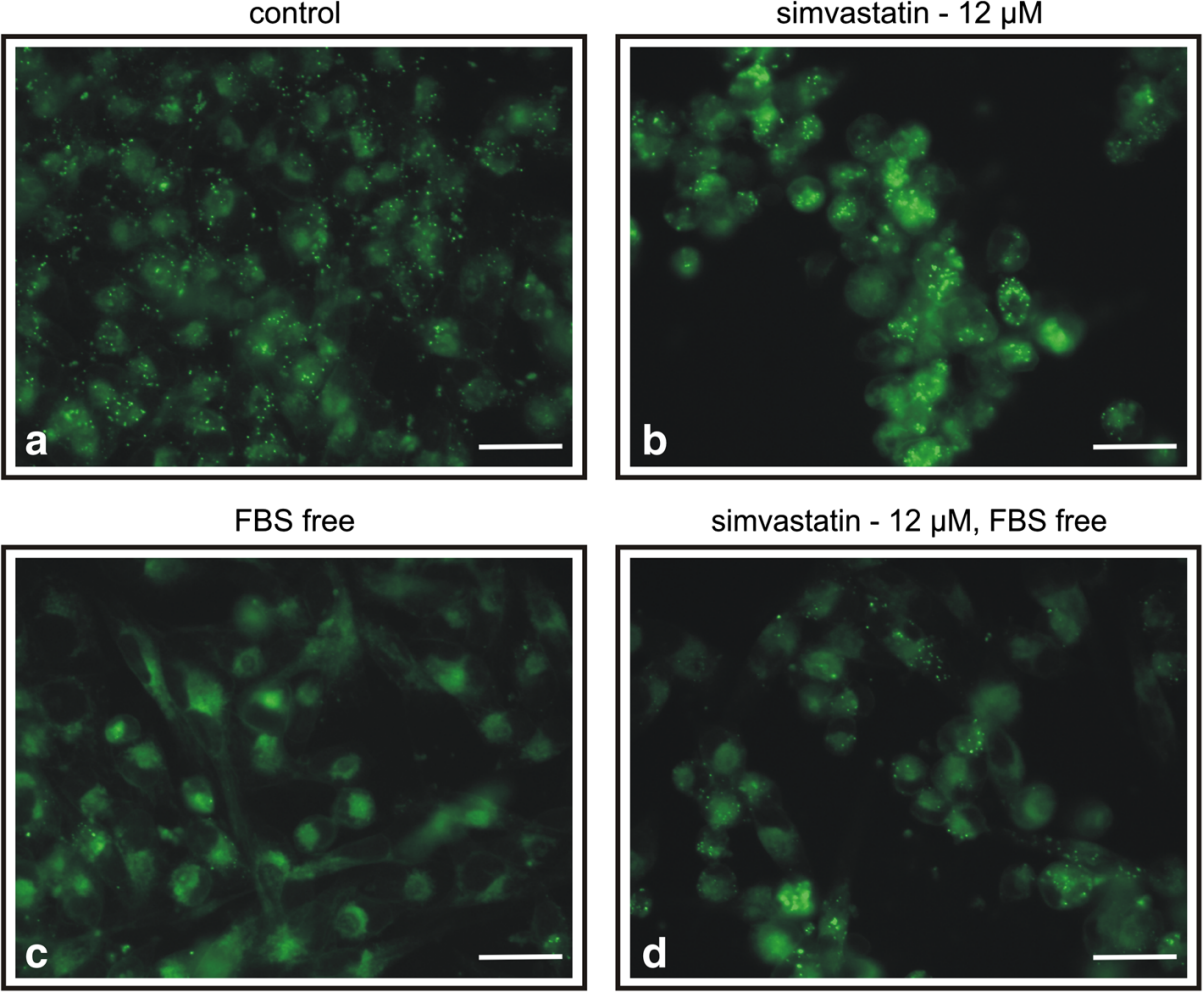
**Intracellular lipid droplets in MiaPaCa-2 cells staining by Nile Red (10 ng/ml), a) control cells, b) simvastatin treated cells, c) cells cultivated in FBS free medium, d) cells treated with simvastatin in FBS free medium.** Simvastatin used in concentration 12 μM, 48 hrs exposure. FBS, fetal bovine serum. Scale bar represents 10 μm.

Content of the LDs in the MiaPaCa-2 cells was in general higher as compared to that in HEK-293T cells (Figure 2a vs. Figure 1a). Due to very high LDs concentration in MiaPaCa-2 cells there were not detectable changes in LDs amounts after 24 hours of treatment. Therefore, the MiaPaCa-2 cells were cultured in the FBS free medium supplemented with 12 μM simvastatin for 48 hours. Even after this prolonged exposure to simvastatin, the changes in the LDs amount in MiaPaCa-2 cells (Figure 2b) were not as evident as in HEK cells. Similarly, the amount of LDs was decreased in the MiaPaCa-2 cells cultured in the FBS free medium compared to those containing FBS (Figure 2c vs. Figure 2a). The LDs content in the MiaPaCa-2 cells treated with simvastatin in the FBS free medium was again slightly higher to that in the cells cultured in the same medium in absence of simvastatin (Figure 2d vs. Figure 2c) and substantially lower compared to that in the control cells (in the FBS supplemented medium) (Figure 2a).

### Analysis of intracellular cholesterol content

To study the composition of accumulated LDs, MiaPaCa-2 cells were exposed to 12 μM simvastatin for 48 hours. The cells were then harvested and cellular cholesterol content was quantified and compared to that in the cells cultured in the absence of simvastatin. Only free cholesterol was detected in the analyzed samples, but no significant differences between cholesterol content in the control cells and those treated with simvastatin were detectable (Table 1).

**Table 1:** Cholesterol content in MiaPaCa-2 exposed to simvastatin

	**Control**	**Simvastatin-treated**	**% Control**	***P-*value**
Total cholesterol [nmol]	97 ± 7	91 ± 6	94%	NS
Free cholesterol [nmol]	98 ± 8	93 ± 7	95%	NS
*P-*value	NS	NS	-	-

Cells were exposed to simvastatin (12 μM) for 48 hrs. Data represent the mean of triplicate determinations ± SD; NS, not significant.

### Gene expression analysis

#### a) The effect of simvastatin on expression of genes related to lipid metabolism

Gene expression microarray analysis revealed that 6 μM concentration of simvastatin was sufficient for up-regulation of three genes related to the enzymes of the mevalonate pathway; this effect was pronounced by higher concentration of simvastatin (12 μM) (Table 2).

**Table 2:** The effect of simvastatin on expression of genes involved in lipid metabolism in MiaPaCa-2 cells

**Gene symbol**	**Product name and related functional pathway**	**RefSeq ID**	**Fold change after simvastatin**		**FDR**
			**6 μM**	**12 μM**	
*HMGCS1*	3-hydroxy-3-methylglutaryl-coenzyme A synthase (EC 2.3.3.10)	NM_002130.6	3.48	3.63	3.3 × 10^-8^
	Mevalonate pathway (cholesterol synthesis)				
*HMGCR*	3-hydroxy-3-methylglutaryl-coenzyme A reductase (EC 1.1.1.34)	NM_000859.1	2.58	3.11	2.1 × 10^-6^
	Mevalonate pathway (cholesterol synthesis)				
*MVD*	Mevalonate pyrophosphate decarboxylase (EC 4.1.1.33)	NM_002461.1	2.31	2.39	3.4 × 10^-7^
	Mevalonate pathway (cholesterol synthesis)				
*PPAP2A*	Phosphatidic acid phosphatase 2a (EC 3.1.3.4)	NM_003711.2	1.79	2.25	3.3 × 10^-5^
	Kennedy pathway (triacylglycerol synthesis)				
*AGPAT2*	1-acyl-glycerol-phosphate acyltransferase 2 (EC 2.3.1.51)	NM_006412.3	1.71	2.01	5.0 × 10^-5^
	Kennedy pathway (phospholipids and glycerolipids synthesis)				
*ACSS2*	Acyl-CoA synthetase short-chain family member 2 (EC 6.2.1.1)	NM_018677.2	2.16	2.22	1.7 × 10^-6^
	Activation of long chain fatty acids				
*ABCA7*	ABC transporter sub-family A member 7	NM_019112.3	1.67	2.03	2.3 × 10^-5^
	Transporter involved in cholesterol and lipid homeostasis				

Simvastatin used in concentration 6 and 12 μM, 24 hrs exposure. Presented are only transcripts with FC > 2.0 or < 0.5 and FDR < 0.05. For full list of differentially regulated transcripts see the ArrayExpress database, accession number E-MTAB-1501.

The most upregulated gene of mevalonate pathway was *HMGCS1* (Table 2) coding for an enzyme catalyzing condensation of acetyl-CoA with acetoacetyl-CoA to form HMG-CoA. The other up-regulated gene, *HMGCR* (3-hydroxy-3-methylglutaryl-coenzyme A Reductase) is responsible for conversion of the HMG-CoA to mevalonic acid and it is target of statins. The MVD (mevalonate pyrophosphate decarboxylase), whose gene transcription was doubled upon treatment with simvastatin, catalyzes conversion of mevalonate pyrophosphate into isopentenyl pyrophosphate [17].

Treatment of simvastatin also lead to significant up-regulation of two genes coding for enzymes catalyzing *de novo* synthesis of phospholipids and glycerolipids, in particular 1-acyl-sn-glycerol-3-phosphate acyltransferase beta (*AGPAT2*) and lipid phosphate phosphohydrolase 1 (*PPAP2A*) (Table 2). AGPAT2 converts lysophosphatidic acid to phosphatidic acid, the second step in *de novo* phospholipid biosynthesis [18]. PPAP2A is a member of the phosphatidic acid phosphatase (PAP) family. PAPs convert phosphatidic acid to diacylglycerol, and function in *de novo* synthesis of glycerolipids as well as in receptor-activated signal transduction mediated by phospholipase D. This protein plays an active role in hydrolysis and uptake of lipids from extracellular space and represents a key enzyme in the regulation of lipid synthesis in general [19].

In addition, *ACSS2* coding for acyl-CoA synthetase short-chain family member 2 (the member of Acyl-CoA synthases (thiokinases)) was also significantly upregulated by simvastatin (Table 2). It catalyzes activation of long chain fatty acids and esterifies them to coenzyme A, before they undergo oxidative degradation to be utilized for synthesis of complex lipids (e.g., TG or membrane lipids), or be attached to proteins as lipid anchors [20].

The last significantly up-regulated gene related to cholesterol and lipid homeostasis was *ABCA7* gene (Table 2). The protein encoded by this gene is a member of the superfamily of ATP-binding cassette (ABC) transporters, that transport a wide variety of substrates across extra- and intracellular membranes, including metabolic products, lipids and sterols, and drugs [21].

#### b) The effect of simvastatin on expression of genes involved in cell proliferation

Besides genes involved in lipid metabolism, and consistent with reported antiproliferative effects of statins, simvastatin treatment affected expression of a large number of genes implicated in cell cycle regulation, DNA replication or cell signaling, including MAP kinase signaling pathway. Simultaneously, the genes involved in apoptosis and autophagy have been found to be differentially expressed (see Table 3, for detailed list of genes, see ArrayExpress database, accession number E-MTAB-1501).

**Table 3:** The effect of simvastatin on metabolic pathways of human pancreatic cancer cells MiaPaCa-2

**Path ID**	**Path name**	**FDR**
hsa00100	Biosynthesis of steroids	1.1 × 10^-12^
hsa04110	Cell cycle	1.4 × 10^-9^
hsa01430	Cell communication	1.1 × 10^-6^
hsa03030	DNA replication	1.2 × 10^-6^
hsa04010	MAPK signaling pathway	2.0 × 10^-4^
hsa00230	Purine metabolism	8.0 × 10^-4^
hsa03430	Mismatch repair	1.0 × 10^-3^
hsa00190	Oxidative phosphorylation	1.0 × 10^-3^
hsa03440	Homologous recombination	1.0 × 10^-3^

Simvastatin used in concentration 6 and 12 μM, 24 hrs exposure. FDR < 0.001 was used as a cut-off value. Data based on KEGG pathway analysis, FDR- false discovery rate. For detailed list of genes, see the ArrayExpress database, accession number E-MTAB-1501.

Validation of gene expression changes was performed using quantitative RT-PCR (see results in Additional file 1).

## Discussion

Present study demonstrates that the exposure of human embryonic kidney cells HEK-293T as well as pancreatic cancer cells MiaPaCa-2 to simvastatin, a competitive inhibitor of HMG-CoA reductase, results in increased accumulation of intracellular LDs. As LDs play role in many important developmental processes including proliferation, apoptosis, growth arrest and necrosis, our data seems to be in line with the effect of statins on lipid metabolism and cancer development [14].

It was previously described that LDs contain predominantly TG and CE [14]. Based on this data, a decrease of intracellular LDs after inhibition of mevalonate pathway by simvastatin was expected. However, the intracellular amount of LDs in both noncancerous HEK 293T as well as malignant pancreatic cancer MiaPaCa-2 cells increased after the exposure to simvastatin.

To account for this phenomenon, we quantified the cholesterol content in studied cells. Surprisingly, the intracellular cholesterol did not differ between statin-exposed compared to untreated cells indicating that accumulation of other lipids must be responsible for our observation.

In fact, the expression analysis revealed up-regulation of multiple genes involved in lipid metabolism. The up-regulated *ABCA7* gene coding for transporter involved in cholesterol and lipid homeostasis is likely to enhance the cholesterol uptake from medium in the cholesterol-depleted cells. This is consistent with the observation that in most cell lines the numbers of LDs are largely determined by the composition of the culture medium [22]. This correlates with the fact that all nucleated cells tightly regulate their intracellular free cholesterol concentrations not only through the endogenous cholesterol synthesis, but also through LDL-receptor and ABC transporters-mediated cholesterol transport [23,24]. Similarly, Iwamoto et al. concluded that cholesterol depletion induced the up-regulation of *ABCA7* gene expression [25].

However, the amount of intracellular LDs increased also in the cells treated with simvastatin in FBS free medium suggesting that *de novo* lipid synthesis must be responsible for this phenomenon in the cells cultured in cholesterol-depleted cultivation medium. Our observations correlate with the conclusion of Williams et al., who observed an accumulation of LDs in keratinocytes cultivated in serum free medium supplemented with lovastatin [26].

As noted before, the second major component of intracellular LDs are TGs. Enhanced TGs synthesis might thus account for the increase of intracellular LDs in the simvastatin-treated cells in an effort to balance constant amount of soluble cytoplasmic fatty compounds while keeping a constant level of cholesterol.

**Figure 3:**
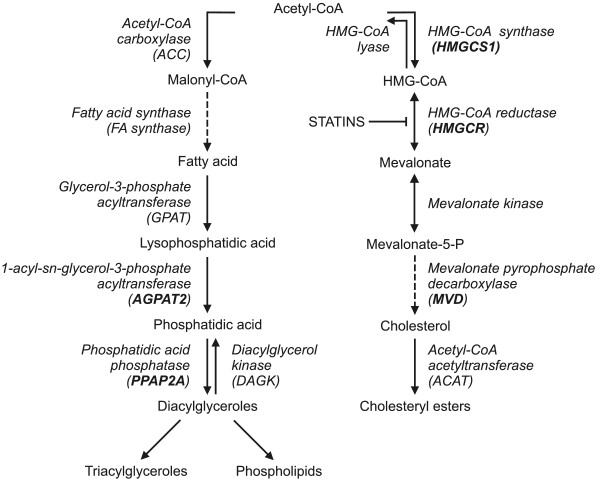
**Scheme of simvastatin-affected metabolic pathways related to lipid metabolism according to our model.** Products of genes affected by simvastatin are shown bold.

Indeed, apart from up-regulation of *ABCA7* lipid transporter gene, simvastatin substantially induced also genes involved in TG and phospholipid *de novo* biosynthesis (Figure 3); which is in accord with the explanation suggested above for the accumulation of LDs in the simvastatin-treated cells.

HMG-CoA synthase, which catalyzes condensation of acetyl-CoA with acetoacetyl-CoA to form HMG-CoA, is up-regulated (Figure 3). The reduction of HMG-CoA to mevalonate by HMGCR is inhibited by the simvastatin; in turn, molecules of HMG-CoA accumulate in the cell and inhibit HMG-CoA synthase by a feedback product inhibition (Figure 3). Consequently, the acetyl-CoA and acetoacetyl-CoA accumulate in the cells providing a high pool of acetyl-CoA that could serve as a basic building component of TG (Figure 3).

We have not observed changes in expression of genes encoding two key enzymes involved in cytosolic fatty acid biosynthesis; namely acetyl-CoA carboxylase (ACC), which converts acetyl CoA to malonyl CoA and fatty acid synthase (FA synthase) (Figure 3) (data not shown). However other genes, namely *PAP2a* and *AGPAT2*, required for *de novo* TG and phospholipids synthesis were upregulated after cells treatment by higher doses of simvastatin (Table 2). These two enzymes catalyze reactions downstream to the rate limiting step of the mentioned routes (Figure 3). The simvastatin induced increase of TGs and phospholipids synthesis may also explain the aforementioned up-regulation of *ABCA7* gene, because ABCA7 modulates not only cholesterol levels but also phospholipid release by apolipoproteins in cell cultures [27-29].

Recently, Boren and Brindle observed that induction of apoptosis resulted in rapid accumulation of cytoplasmic LDs and was accompanied by an increase of *de novo* neutral lipid synthesis, most likely due to the inhibition of mitochondrial fatty acid β-oxidation. They showed that the formation of cytoplasmic LDs was caused by an apoptosis-induced mitochondrial dysfunction [30], a phenomenon affected by simvastatin also in our studies.

Simultaneously, simvastatin was able to modulate expression of a wide array of genes implicated in cell cycle regulation and signaling. It remains to be answered whether these effects are independent or are functionally associated with the accumulation of LDs. As LDs are supposed to be derived from the ER membrane [12,14] that serves as a pool of many enzymes required for lipid metabolism as well as the programmed cell death [30,31], accumulated intracellular LDs in statin-treated cells might be rather effectors of some pleiotropic effects of statins than only by-standing phenomenon with no biological relevance.

In conclusion, simvastatin treatment led to accumulation of cytosolic LDs within both non-cancerous as well as malignant cells, a phenomenon which might contribute to their antiproliferative effects.

## Methods

### Cell cultures and simvastatin treatment

Human embryonic kidney (HEK-293T) and pancreatic cancer cells (MiaPaCa-2, both cell lines from ATCC, Manassas, VA, USA) were maintained and grown in a humidified atmosphere containing 5% CO_2_ at 37°C in DMEM supplemented with 10% fetal bovine serum. Cell viability was assessed by 0.4% trypan blue staining. Simvastatin was added at concentrations equal to its IC50 value for MiaPaCa-2 (12 μM) as reported previously [10] 24 hrs post-inoculation of the cells. For DNA microarray analyses also 6 μM concentrations of simvastatin were used. For all studies, pure form of simvastatin was used (Enzo Life Sciences, NY).

### Lipid droplets staining

Two neutral lipid dyes, Nile Red (9-diethylamino-5H-benzo[α]phenoxazine-5-one) and BODIPY® 493/503 (4,4-difluoro-1,3,5,7,8-pentamethyl-4-bora-3α,4α-diaza-s-indacene) were used for lipid staining. The stock solutions (1.0 mg/ml) in methanol were stored frozen (−20°C) in dark. Staining was carried out in fixed cells (4% formaldehyde, 20 minutes). The dye was added directly to the fixed cells to a final concentration of 10 ng/ml and incubated for 10 minutes. The dye was carefully washed out using PBS prior to microscopy. LDs were then visualized by fluorescence microscopy using QuickPHOTO CAMERA 2.1 processing software (Olympus, Tokyo, Japan).

### Quantification of cellular cholesterol content in MiaPaCa-2 cells exposed to simvastatin

The cellular cholesterol content in MiaPaCa-2 cells exposed to simvastatin (12 μM) [10] 24 hrs post-inoculation of MiaPaCa-2 cells were quantified using isotope dilution-gas chromatography–mass spectrometry (ID-GC-MS). The amounts of the total (free cholesterol and cholesterol ester) and free cholesterol were analyzed after additional 48 hrs of incubation (in triplicate) to determine the amount and form of cholesterol present in the cells exposed to simvastatin. The cell pellet was mixed with D6-cholesterol (internal standard, 50 mg/l) (Medical Isotopes, Inc, Pelham, AL, USA), resuspended in ethanol with 9 M KOH and incubated at 37°C for 3 hrs for complete hydrolysis of cholesterol esters. The samples were then diluted with water and extracted into hexane. The organic phase was evaporated at 60°C under a stream of nitrogen. Dried samples were reconstituted in N,O-bis(trimethylsilyl)acetamide (TMA) (Merck) and silylated 30 minutes at room temperature. Relative concentrations of resulting cholesterol trimethylsilylethers were determined using gas chromatography (Agilent 6890, Agilent, USA) coupled with a quadrupole mass detector (Agilent 5973, Agilent, USA). Analytical conditions were: splitless injection, inlet temperature 300°C, column HP 5MS, temperature gradient from 200°C to 300°C - 10°C/min, column flow 1 ml/min, electron impact ionization at 70 mV, single ion monitoring at 458.4 and 464.4. Absolute intracellular cholesterol levels were calculated from calibration curves using purified cholesterol standard (Sigma-Aldrich, USA). The cholesterol content of simvastatin treated MiaPaCa-2 cells was normalized to the cell number in the sample.

Cholesterol content is expressed as mean ± SD. The differences between values acquired were evaluated by Student’s t-test. Differences were considered statistically significant when p values were less than 0.05.

### DNA microarray analysis

The effect of 6 and 12 μM concentrations of simvastatin on MiaPaCa-2 pancreatic cancer cell gene expression was investigated 24 h post-inoculation. Cells from two parallel cultivations (10 cm^2^ cultivate dish) were lysed in the stage of subconfluency using the RLT lysis buffer supplied in RNeasy Mini Kit (Qiagen, USA). Total RNA was isolated by RNeasy Micro Kit (QIAGEN, USA) according to the procedure for animal cells. Quantity of the RNA was measured on NanoDrop ND-1000 spectrophotometer (NanoDrop Technologies LLC, USA). Quality of the RNA was analyzed on Agilent 2100 Bioanalyser (Agilent Technologies, CA, USA). The RNA samples had RIN (RNA integrity number) above 9.

Illumina Human-6 v2 Expression BeadChip (Illumina, USA) was used for the microarray analysis following the standard protocol. Total RNA (150 ng) was amplified using Illumina TotalPrep RNA Amplification Kit (Ambion, USA) and 15 microgram of amplified RNA was hybridized on the chip according to the manufacturer’s protocol. All subsequent analyses were done on two biological replicates.

The raw data (TIFF image files) was analyzed with *beadarray* package [32] of the *BioConductor* within the *R* environment [33]. All hybridizations passed quality control. The data was background corrected and normalized with the probe level quantile method. Before detection of differential expression, the probes with intensity level lower than 95-percentile of negative controls of the BeadChip in all samples were disregarded from the analysis. Probes with low variation across samples estimated by the inter-quartile range irrespective of the sample group were disregarded as well. Differential expression was detected using the moderated t-test of the *limma* package [34] on intensities that were variance-stabilized by logarithmic transformation. Annotation of the transcripts differentially expressed between simvastatin treated and the control cultures was done using the manifest file provided by the manufacturer (HumanWG-6_V2_0_R2_11223189_A.bgx; Illumina, USA). The transcripts with false discovery rate (FDR) [35] smaller than 0.05 and fold change smaller than 0.5 or higher than 2 were reported and used in the downstream analysis. The results were deposited to the ArrayExpress database under accession number E-MTAB-1501. Further, we performed the gene set enrichment analysis (GSEA) on KEGG pathways [36] using the Fisher’s exact test and the approach of Tian [37].

### Quantitative real-time PCR

Reverse transcription was performed by QuantiTect Reverse Transcription Kit (QIAGEN Inc., USA). The qRT-PCR was performed on LightCycler 2.0 System using LightCycler 480 DNA SYBR Green I Master kit (Roche Diagnostics, Germany) and results were analyzed by LightCycler software. Crossing point values were further determined using the R environment [33]. Detailed description of the analysis and the list of amplicons/primers of target and housekeeping genes are provided in ArrayExpress database, accession number E-MTAB-1501.

## Competing interests

The authors declare that they have no competing interests.

## Authors’ contributions

Conceived and designed the experiments: HG, LV, TR, MŠ. Performed microscope experiments: MŠ, IV, LL, HG. Performed HPLC: JZ. Performed gene expression analysis: HS, MK. Analyzed, interpreted the data and drafted manuscripts: HG, TR, LV, DB. All authors read and approved the final manuscript.

## Supplementary Material

Additional file 1 Validation of gene expression changes performed using quantitative real-time polymerase chain reaction (qRT-PCR). Click here for file
