# Supplementary material for: The effect of simvastatin on lipid droplets accumulation in human embryonic kidney cells and pancreatic cancer cells
Source: Lipids Health Dis. 2013 Aug 21;12:126. doi: 10.1186/1476-511X-12-126 (PMC3765626; doi:10.1186/1476-511X-12-126)
Supplement: Supplementary file 1 [file 1476-511X-12-126-S1.doc]

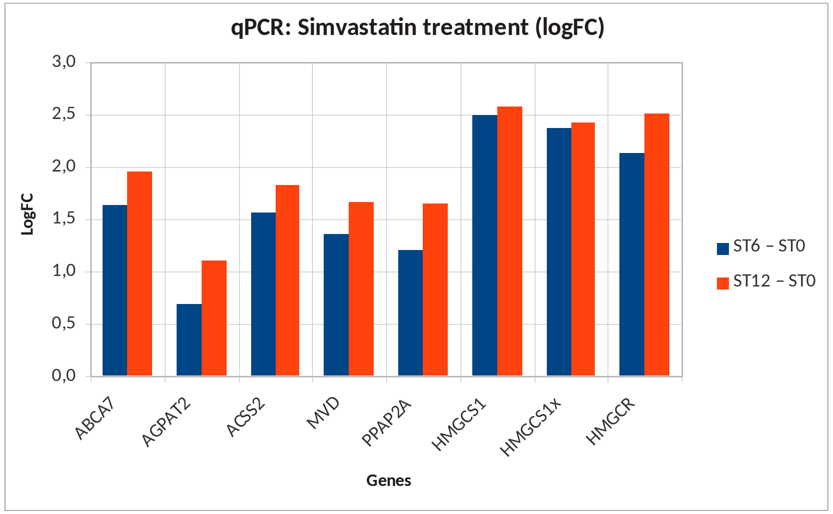
qPCR: Simvastatin treatment (logFC)
3,0
2,5
2,0
1,5
1,0
0,5
0,0
LogFC
ABCA7
AGPAT2
ACSS2
MVD
PPAP2A
HMGCS1
HMGCS1x
HMGCR
Genes
ST6 – ST0
ST12 – ST0

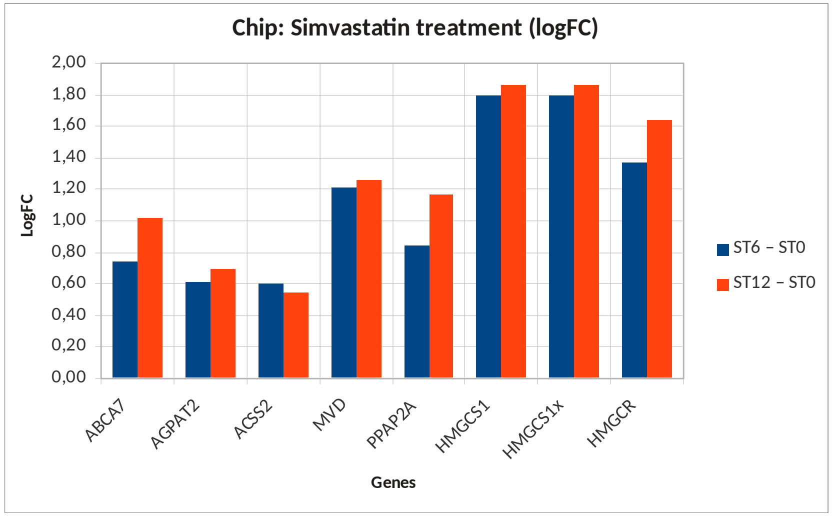
Chip: Simvastatin treatment (logFC)
2,00
1,80
1,60
1,40
1,20
1,00
0,80
0,60
0,40
0,20
0,00
LogFC
ABCA7
AGPAT2
ACSS2
MVD
PPAP2A
HMGCS1
HMGCS1x
HMGCR
Genes
ST6 – ST0
ST12 – ST0
